# Maxwell A. Pollack Award for Contributions to Healthy Aging Presentation and Lecture

**DOI:** 10.1093/geroni/igaf122.670

**Published:** 2025-12-31

**Authors:** Jung Kwak

## Abstract

The Maxwell A. Pollack Award for Contributions to Healthy Aging Lecture will feature an address by the 2024 Pollack Award recipient Edward Alan Miller, PhD, MPA, FGSA, of the University of Massachusetts Boston. This session will also include the presentation of the 2025 Maxwell A. Pollack Award to recipient John W. Rowe, MD, FGSA, of Columbia University. The Maxwell A. Pollack Award for Contributions to Healthy Aging Award recognizes instances of practice informed by research and analysis, research that has directly improved policy or practice, and distinction in bridging the worlds of research and practice.

